# Lung neuroendocrine neoplasms: recent progress and persistent challenges

**DOI:** 10.1038/s41379-021-00943-2

**Published:** 2021-10-18

**Authors:** Natasha Rekhtman

**Affiliations:** Department of Pathology, Memorial Sloan Kettering Cancer Center, 1275 York Ave, New York, NY 10065 USA

**Keywords:** Small-cell lung cancer, Non-small-cell lung cancer

## Abstract

This review summarizes key recent developments relevant to the pathologic diagnosis of lung neuroendocrine neoplasms, including carcinoids, small cell lung carcinoma (SCLC), and large cell neuroendocrine carcinoma (LCNEC). Covered are recent insights into the biological subtypes within each main tumor type, progress in pathological diagnosis and immunohistochemical markers, and persistent challenging areas. Highlighted topics include highly proliferative carcinoids and their distinction from small cell and large cell neuroendocrine carcinomas (NECs), the evolving role of Ki67, the update on the differential diagnosis of NEC to include thoracic SMARCA4-deficient undifferentiated tumors, the recent data on SCLC transcriptional subtypes with the emergence of POU2F3 as a novel marker for the diagnosis of SCLC with low/negative expression of standard neuroendocrine markers, and the update on the diagnosis of LCNEC, particularly in biopsies. There has been remarkable recent progress in the understanding of the genetic and expression-based profiles within each type of lung neuroendocrine neoplasm, and it is hoped that these insights will enable the development of novel diagnostic, prognostic, and predictive biomarkers to aid in the pathologic assessment of these tumors in the future.

## Introduction

In the last decade, there has been a tremendous progress in identifying molecular subtypes in non-small cell lung carcinoma (NSCLC), forming the basis of increasingly personalized systemic therapies for this disease. Conversely, until recently, there has been only limited progress in the identification of clinically relevant biological subsets and the development of personalized approaches in the field of lung neuroendocrine (NE) neoplasms. However, in recent years, there has been several significant breakthroughs in understanding of the molecular characteristics of each major type of lung NE neoplasm, which may set the stage for significant progress in the near future. This article will address the areas of recent progress and persistent challenges in pulmonary NE neoplasms, focusing on the areas relevant to pathologic diagnosis.

### Thoracic WHO classification 5^th^ edition (2021): terminology and criteria for neuroendocrine neoplasms

The criteria and terminology for lung NE neoplasms in the latest World Health Organization (WHO) classification of thoracic tumors (5^th^ edition; 2021) remains largely unchanged from the prior edition^1,2^. Major WHO NE tumor types include typical carcinoid, atypical carcinoid, small cell lung carcinoma (SCLC), and large cell NE carcinoma (LCNEC). In this article, the term NE carcinoma (NEC) will be used as an umbrella term for SCLC and LCNEC. Combined carcinomas are defined as tumors with both NEC and NSCLC components.

Overall, NE neoplasms account for ~20% of lung primary tumors, with SCLC accounting for ~15%, carcinoids ~2% (with typical to atypical ratio of 10:1), and LCNEC ~3%. Notably, over the last few decades, the incidence of SCLC has decreased significantly in the United States largely as a result of decreased cigarette smoking, whereas the incidence of carcinoids has substantially increased, likely due to increased use of Computed Tomography (CT) imaging. The incidence of LCNEC has also increased, which may reflect greater recognition of this entity^1^.

The defining WHO criteria for typical carcinoid vs atypical carcinoid vs NEC are based primarily on mitotic counts per 2 mm^2^; the presence of necrosis (usually focal/punctate, comedo-like) is an additional criterion for atypical carcinoid (Table 1). It is important to recognize, however, that while the mitotic criteria for typical vs atypical carcinoids represent grades of the same disease entity, the mitotic criteria for carcinoids vs NEC does NOT represent grades of the same disease. Instead, these are biologically distinct entities, as supported by recent molecular studies indicating sharply distinct genomic profiles of these tumors^3^. Thus, the major distinguishing feature for carcinoids vs NEC is fundamentally their distinct overall morphology, whereas distinct proliferation rates is a characteristic property but not the sole defining feature. This concept is increasingly important given the recognition of a proliferative gray-zone between some carcinoids and NECs, as will be discussed below.

**Table 1:** The thoracic versus digestive WHO terminology and criteria for neuroendocrine neoplasms.

Thoracic WHO 5th Ed, 2021		Digestive WHO 5th Ed, 2019		
Terminology	Criteria: Mitotic counts per 2 mm^2^	Terminology	Criteria:	
			Mitotic counts per 2 mm^2^	Ki67 index
Typical carcinoid	<2	NET, grade 1	<2	<3%
Atypical carcinoid	2–10 (or necrosis)	NET, grade 2	2–20	3–20%
–	–	NET, grade 3	>20	>20%
SCLC and LCNEC (NEC)	>10	NEC (small cell or large cell)	>20	>20%
Combined NEC and NSCLC		MiNEN		

*LCNEC* large cell neuroendocrine carcinoma, *MiNEN* mixed neuroendocrine-non-neuroendocrine neoplasm, *NEC* neuroendocrine carcinoma, *NET* neuroendocrine tumor, *NSCLC* non-small cell lung carcinoma, *SCLC* small cell lung carcinoma.

Of note, 2 mm^2^ in practice is commonly measured as 10 high-power fields (HPFs), where HPF is a field of view with a 40x objective. However, the actual number of HPFs is microscope-dependent, and in many modern microscopes, 2 mm^2^ corresponds to ~8.5 HPFs^4^. It is recommended that for values near the thresholds (2 per 2 mm^2^ for typical vs atypical carcinoids, and 10 per 2 mm^2^ for carcinoids vs NEC) counts should be averaged from 3 sets of 2 mm^2^ fields in the areas of highest proliferative activity^1^.

### Pulmonary vs digestive tract NE neoplasms

The comparison of the terminology and criteria for NE neoplasms of lung versus digestive tract (tubular GI tract and pancreas)—the second largest site of origin of NE neoplasms in the body after the lung—is summarized in Table 1^5^. In the digestive tract, the term carcinoid has been replaced with the term “NE tumor” (NET). Although lung carcinoids belong to the general family of NETs, it is recommended by the thoracic WHO that the main diagnostic term for lung tumors remain “carcinoid” (or alternatively “carcinoid/NET”) for the clarity of communication with the thoracic treating physicians.

While Ki67 is currently a standard marker for grading digestive NETs as G1 (low-grade) vs G2 (intermediate-grade) vs G3 (high-grade), the distinction of typical vs atypical carcinoids (corresponding to G1 and G2, respectively) in the lung still relies on mitotic counts only. However, the diagnostic and prognostic value of Ki67 for lung carcinoids is increasingly recognized and the category comparable to G3 NETs is also emerging (see below).

Lung carcinoids overall account for ~30% of NETs in the body. Notably, the vast majority of lung carcinoids are early-stage tumors, and only a minority of patients develop distant metastases^1,2^. Conversely, the prevalence of metastatic disease is substantially higher for pancreatic and tubular GI NETs, including at presentation^6^. Therefore, the vast majority of pathologic specimens from lung carcinoids are lung tumor resections, and—unlike digestive NETs—only a small proportion of specimens is from metastatic tumors. As a result, pathologic criteria for lung carcinoids are primarily geared toward the prediction of recurrence/metastasis for resected primary tumors, whereas in digestive NETs—the criteria are more adapted to small biopsies and metastatic specimens.

The distribution of metastatic sites is also significantly different for NETs of lung vs digestive organs. While both types commonly metastasize to the liver, lung carcinoids also frequently metastasize to bone and brain, whereas such metastatic sites are distinctly uncommon for digestive NETs^7,8^. This can help guide the differential diagnosis when considering the site of origin of metastatic NET of unknown primary.

SCLC accounts for >90% of small cell carcinomas in the body, with the esophagus, bladder, cervix, and anus being a distant second^9^. Notably, classic pulmonary-type small cell carcinomas are distinctly uncommon in tubular GI tract and pancreas, where most NECs are of large cell type^10,11^.

### Update on neuroendocrine markers

Traditional NE immunohistochemical (IHC) markers that are widely used in pathology practice include synaptophysin, chromogranin A, and CD56 (NCAM1). Neuron-specific enolase (NSE) is not recommended due to low specificity. While virtually all carcinoids are positive for synaptophysin and chromogranin A, their expression is highly variable in NECs. It is well known that up to 15–20% of SCLC are negative for both synaptophysin and chromogranin A, but most of such tumors are positive for CD56. Therefore, while CD56 is generally not recommended as a NE marker at non-pulmonary sites due to its lower specificity, it has been widely used in thoracic pathology for the diagnosis of SCLC, especially prior to the wide use of Insulinoma-associated protein 1 (INSM1)^12^. However, undoubtedly, CD56 should be used with caution if positive in isolation since it can be strongly expressed by a variety of non-NE neoplasms, including various hematolymphoid neoplasms, such as NK/T cell and other T-cell lymphomas^1,12^.

In the last decade, INSM1 has emerged as a novel and useful NE marker. It is a pan-neuroendocrine marker, expressed in NETs and NECs of all sites, including lung carcinoids, SCLC, and LCNEC^13–15^. INSM1 is a nuclear transcription factor, offering an advantage in interpretation over prior markers which are all cytoplasmic. Similar to CD56, INSM1 is positive in many SCLC lacking expression of synaptophysin and chromogranin A^15^. However, recent studies show that INSM1 can also be expressed in a variety of non-NE neoplasms, including soft tissue sarcomas, where INSM1 is detected generally more commonly than other NE markers^16–18^. While expression in some sarcoma may reflect true NE differentiation, INSM1 positivity has also been documented in some clearly non-NE tumors like adenoid cystic carcinoma and even lymphoma^17^. While it was initially suggested that INSM1 could serve as a standalone NE marker^13^, given the growing recognition of its wider expression and the need for more studies to assess the relative contribution of other markers, currently INSM1 is best regarded as a useful addition to NE marker panel rather than a standalone replacement of the existing markers.

Achaete-scute homolog-like 1 (ASCL1, also known as hASH1 or MASH) is another neurogenic/NE transcription factor, which has been long known to pathologists as a robust IHC marker. Although highly specific, it has significantly lower sensitivity than INSM1 since it is expressed only in a subset of NECs and carcinoids^19^. It has emerged recently as a key transcriptional subtype marker in SCLC, but its routine diagnostic role is not established.

## Carcinoids

### Morphologic spectrum

The prototypical morphologic features of lung carcinoids are those of well-differentiated NETs in general. This includes bland, uniform round or plasmacytoid cytology, smooth nuclear membranes, granular/speckled (salt and pepper) chromatin, absent or only subtle nucleoli, and various architectural features typical of NETs (nesting/organoid pattern, cords/trabeculae, and rosettes). Lung carcinoids may exhibit a staggering number of variant morphologies, which can cause diagnostic difficulties, especially in biopsies. Most notably, this includes the acinar pattern—as commonly seen in digestive NETs—which can closely mimic adenocarcinoma, and spindle cell pattern, which is particularly common in the peripherally located carcinoids, and can mimic various low-grade spindle cell neoplasms, such as a solitary fibrous tumor or synovial sarcoma^2^. Oncocytic features and the presence of papillary architecture can also cause diagnostic challenges^2^.

### Highly-proliferative carcinoids: an emerging variant

According to the thoracic WHO, a mitotic count of 10 per 2 mm^2^ is defined as the absolute ceiling rate for atypical carcinoids, and tumors exceeding this threshold, even if only mildly, are by default classified as LCNEC. Similarly, although Ki67 is not currently used as a thoracic WHO criterion, the Ki67 rate of 20–30% (30% in the current WHO) is generally regarded as a ceiling rate for lung carcinoids. This is analogous to the former digestive WHO criteria, where any NET (i.e., tumors with well-differentiated NET morphology) exceeding mitotic counts of 20 per 2 mm^2^ or Ki67 of 20% were classified as NEC. Currently, such tumors are recognized as grade 3 NET^5,20^. Analogous category does not formally exist in the current thoracic WHO, but the existence and characteristics of such tumors are becoming increasingly recognized in the literature. Provisionally, the suggestion in the current WHO is to classify such tumors as “LCNEC with morphologic features of carcinoid tumor”, with the anticipation that optimal classification will be clarified as more data accumulates^1^.

At the time of writing, there has been nearly a dozen recent publications, mostly small series, describing lung tumors with morphology of carcinoids but mitotic counts of >10 per 2 mm^2^. Such tumors also commonly exhibit elevated Ki67 rates, which may exceed 20–30% (see Hermans et al.^21^ and references therein). In most cases, mitotic counts and Ki67 exceed these thresholds only mildly, but a hot-spot Ki67 rates of up to ~60% has been reported. Molecular data available for several of such tumors support their relationship with carcinoids based on the presence of *MEN1* mutations (typical of carcinoids) and the absence of *RB1* and *TP53* alterations (typical of NECs)^7,22^. Limited clinical data also support their similarity with carcinoids rather than NEC in terms of progression and response to therapy. Nevertheless, these tumors appear to be highly aggressive, with 11 of 12 patients in one study developing post-surgical recurrence^23^, as compared to ~20–30% recurrence rate for conventional atypical carcinoids^1,2^.

Notably, highly proliferative carcinoids are uncommon in the primary lung resections, in part explaining the slow accumulation of data on such tumors. Conversely, such tumors are remarkably common in the stage IV setting, where in a recent study, mitotic counts of >10 per 2 mm^2^ and hot-spot Ki67 of >20% were encountered in 23 and 27% of metastatic samples, respectively^7^. In patients with matched primary and metastatic samples, the escalation of both mitoses and Ki67 was observed in a significant proportion of metastatic samples^7,24^, analogous to the observations in digestive NETs^25^. Thus, it is important to be aware of a wide proliferative range in carcinoids in the metastatic setting. If using Ki67 to aid the diagnosis, especially in the metastatic specimens, the rates of >20–60% should be regarded as a “gray zone” as they can occur in both carcinoids and NECs.

**Fig. 1:**
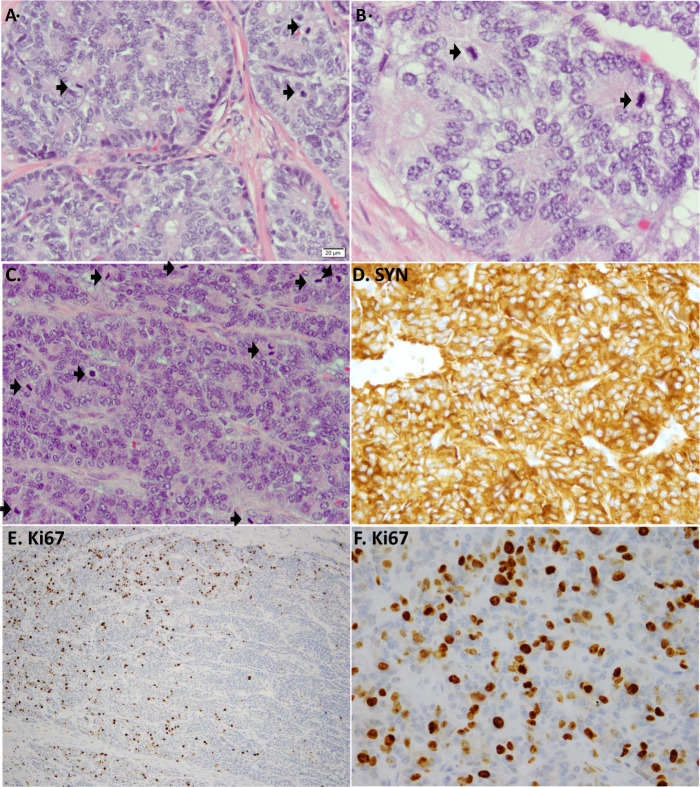
Example of a highly proliferative carcinoid (current WHO terminology “LCNEC with morphologic features of carcinoid tumor”). Example from 2 tumors (**A**, **B** tumor 1, **C**–**F** tumor 2). H&E images show cytologically land tumors, with uniform nuclei and smooth nuclear membranes, typical of carcinoids/NETs, but with strikingly elevated mitotic counts indicated by arrows (>10 per 2 mm^2^). Synaptophysin (SYN; **D**) is strongly and diffusely positive. Ki67 is heterogenous at low-power (**E**) but in hot-spot areas exceeds 30% (**F**). Case 1 corresponds to case ID LCNEC-36 in Rekhtman et al.^22^, which was found to harbor an *MEN1* mutation.

In most cases, highly proliferative carcinoids can be readily recognized morphologically as belonging to the carcinoid/NET family due to their bland, uniform cytology, absence of necrosis (or presence of only focal punctate necrosis), and heterogeneous pattern of Ki67 elevation with hot-spots interspersed with low-proliferative areas (Fig. 1). However, similar to the observation in the digestive NETs, some biopsies from metastases known to be arising from lung carcinoids (based on the tissue diagnosis from the primary tumor) may have increased cell crowding, cytologic atypia, and disorganization, which may present a diagnostic challenge if sampled in isolation^20^. For pancreatic tumors, several ancillary IHC markers are used in this setting, with the loss of Rb and aberrant p53 (block positive or completely lost) supporting NEC, while the loss of DAXX and ATRX supporting NET^20,26^. Analogously, in the lung, the loss of Rb and aberrant p53 can be used to support the diagnosis of NEC, given that these alterations are largely restricted to those tumors^7,27^. However, in contrast with pancreatic NETs, there are no established surrogate IHC markers for genomic alterations typical of lung carcinoids. Comprehensive next-generation sequencing may be informative in such cases, with the finding of the low mutation burden, *MEN1* or *EIF1AX* mutations, and the lack of *RB1* and *TP53* alterations being supportive of carcinoid tumor diagnosis^27^.

### The evolving role of Ki67 in the diagnosis and clinical management of lung carcinoids

Currently, Ki67 is a standard and routine marker in the reporting of all digestive NETs. The role of Ki67 in those tumors is both prognostic and predictive, in that selection of systemic therapies is in part based on Ki67 rate^5,28^. Conversely, for lung carcinoids, the utility and the role of Ki67 continues to be a matter of debate. Nevertheless, there is emerging evidence and practical rationale for the routine inclusion of Ki67 in the reporting of lung carcinoids. This emerging role is reflected in the current thoracic WHO recommendation that even though Ki67 is not required for the classification of carcinoids into typical vs atypical categories, reporting of Ki67 as an additional variable is “desirable”^1^. Routine reporting of Ki67 is also recommended in the guidelines by the European and North American NET societies (ENETS and NANETS)^29,30^. Overall, based on the points summarized below, Ki67 is likely to become a routine marker in the assessment of lung carcinoids, similar to the digestive NETs. While in digestive NETs, there are specific recommendations for Ki67 technical and interpretive assessment (MIB1 clone, scoring 500+ cells in hot-spots)^28^, such standardization will require further studies in lung tumors.

Below is a summary of the potential or established roles of Ki67 in 3 different settings for lung carcinoids:

### Assessment of Ki67 in primary carcinoids

In the resected primary lung carcinoids, there is rarely a diagnostic role for Ki67 as the distinction of carcinoids from NECs can be readily made based on morphologic parameters. Conversely, multiple studies have demonstrated that similar to mitotic counts, Ki67 is a strong prognostic marker in carcinoids, associated with the rate of postoperative recurrence/metastasis^31–34^. Studies differ on the relative predictive value of mitoses vs Ki67, but several studies do suggest added prognostic value of Ki67 beyond the WHO categories^31,33,35^. In particular, studies show that Ki67 of >5% in typical carcinoids^33,36,37^ and >10% overall^24,31^ are associated with adverse prognosis. Importantly, from a practical perspective, the scoring of Ki67 is substantially less time-consuming and more reproducible than the scoring of mitoses^38,39^. Lastly, while the diagnosis of typical vs atypical carcinoids is based on mitotic counts, elevated Ki67 rates (especially 10% or more) are virtually restricted to atypical carcinoids, and could thus prompt a close re-count of mitotic figures if discrepant.

In biopsies from primary carcinoids, assessment of Ki67 for prognosis is usually not needed since—with only rare exception—such tumors undergo surgical resection. Interestingly, several recent studies suggest the potential utility of proliferative assessment of core or bronchoscopic biopsies in guiding the extent of resection, with parenchyma-sparing (wedge or segmentectomy) approaches advocated for low-proliferative/typical carcinoids vs anatomic resection (lobectomy) advocated for more proliferative/atypical carcinoids^40^. Based on a biopsy, mitotic counts alone poorly predict final tumor classification^41^. However, Ki67—even though also subject to sampling—has better correspondence with resected tumors^42^. Overall, the role of proliferative assessment of biopsies in surgical planning remains investigational, but Ki67 is more likely to be useful in this setting than mitotic counts.

### Assessment of Ki67 in metastatic carcinoids

Given that stage IV carcinoids are uncommon, the therapeutic approaches for these tumors are largely extrapolated from the digestive NET, where Ki67 is included as part of parameters guiding treatment selection^30,43,44^. Therefore, Ki67 may be requested by medical oncologists for patients with metastatic lung carcinoids. Conversely, mitotic counts have only a limited role in biopsies given that much larger tissue volume is needed for accurate mitotic counts than Ki67.

### Assessment of Ki67 for the diagnosis of carcinoids vs NECs in biopsies and FNAs

**Fig. 2:**
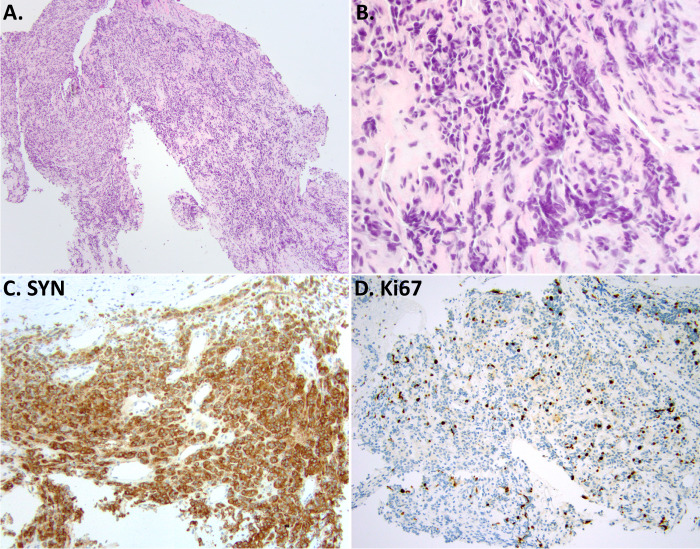
Ki67 for the diagnosis of carcinoid in a crushed biopsy. Low-power (**A**) and high-power (**B**) images of bronchoscopic biopsy, where crush artifact leads to the appearance of cell molding, and together with positive synaptophysin (SYN; **C**) closely mimics the appearance of SCLC. Mitoses are not evaluable in such specimens, but the low Ki67 index (<5%) readily identifies this as a crushed carcinoid rather than SCLC. This remains a common diagnostic pitfall.

One of the most common challenges in the diagnosis of lung NE neoplasms is that of bronchoscopic biopsies, where significant crush artifact can cause carcinoids to have the appearance closely mimicking SCLC (Fig. 2). In this setting, mitotic counts are difficult to assess and the critical role of Ki67 is well established^1,2,45,46^. Most carcinoids should have low rates (1–20%, usually <10%), while most NECs have Ki67 of >50%^45,46^. Although the issue of over-diagnosis of crushed carcinoids as SCLC is well-documented in pathology literature, in the author’s experience, this remains one of the most common pitfalls in practice.

The same diagnostic challenge applies to the cell blocks of fine-needle aspirates (FNAs). Here an additional consideration is that some alcohol fixatives can significantly inhibit the reactivity of the widely used Ki67 clone MIB1^47^. Therefore, caution should be exercised in interpreting Ki67 in such specimens.

### Other potential prognostic markers in lung carcinoids

Orthopedia homeobox protein (OTP) is a neurodevelopmental transcription factor expressed exclusively in lung carcinoids but not NEC, NSCLC, or NETs of non-pulmonary origin, with rare exceptions^48–50^. Thus, it has been suggested to be a useful marker of lung origin for metastatic NETs. In addition, OTP—along with the adhesion molecule CD44—have been suggested to represent strong favorable prognostic markers in lung carcinoids, independent of WHO categories^49^. However, their clinical utility requires further validation.

### Reporting of carcinoids in biopsies and FNAs

A novel aspect for carcinoid tumors in the latest thoracic WHO edition is the clarification of the approach specifically to small biopsies and FNAs. It is clarified that typical vs atypical categories are intended as grades for resected primary tumors to predict the risk of post-surgical recurrence, whereas mitotic-based grading has limited feasibility and accuracy in non-resection specimens. In metastatic setting, separating typical from atypical carcinoids is further compounded by temporal and spatial heterogeneity of proliferation rate at different metastatic sites and different time-points of disease progression^7^. It is therefore now recommended to document tumor type in non-resection specimens as “carcinoid tumor, not otherwise specified” and report the evident mitotic counts and Ki67 proliferation rate without classifying tumors as typical vs atypical^1^. However, if elevated mitotic counts and/or punctate necrosis are evident in the biopsy, the diagnosis of atypical carcinoid can be readily suspected.

### IHC for carcinoids: an update

Carcinoids are consistently and diffusely positive for all standard NE markers (synaptophysin, chromogranin A, CD56) and INSM1. Weak or absent labeling for NE markers, especially if several, can serve as a soft feature favoring NECs over carcinoids. TTF-1 is positive in ~30–50% of carcinoids and can serve as a marker of lung origin in a metastatic setting (unlike small cell or large cell NECs, where TTF-1 is expressed irrespective of the site of origin^2,12^). As mentioned above, OTP is suggested as another marker of pulmonary origin for carcinoids^51^, although the actual prevalence of OTP-positive carcinoids in metastases is unclear given their suggested distinctly indolent nature.

### Emerging biological subtypes of carcinoids

Although lung carcinoids have been regarded pathologically as a single entity, 2 recent multi-omic studies suggest that these tumors comprise 3 distinct biological subtypes (of which 2 may be related) based on their highly distinct gene expression, methylation, and mutation profile^27,52^. These do not strictly correlate with typical vs atypical categories but are significantly associated with central vs peripheral location. Of these, the subtype with *MEN1* mutations and low expression of *OTP* (Cluster B) was associated with poor prognosis^27^, in line with other studies on these individual markers. In addition, a small group of tumors designated “supra-carcinoids” was described as having “carcinoid-like morphology yet the molecular and clinical features of NEC”^27^; further studies will be needed to clarify clinicopathologic features of such tumors. Notably, the existence of distinct subtypes of carcinoids has been suggested in prior electron microscopy^53^ and pathologic studies of carcinoids^54^. Overall, these data provide insights into the underlying biology and possibly distinct cells of origin of lung carcinoids, but diagnostic and clinical relevance for routine practice remains to be determined.

## Diffuse idiopathic pulmonary neuroendocrine cell hyperplasia: an update

Diffuse idiopathic pulmonary neuroendocrine cell hyperplasia (DIPNECH) is a fairly common but still under-recognized condition^1,2^. It manifests pathologically as the presence of multiple bilateral carcinoid tumorlets (<0.5 cm size) and NE cell hyperplasia (increased number intrabronchial NE cells singly or in clusters). In this background, there are commonly full-grown carcinoid tumors, distinguished from tumorlets by being ≥0.5 cm in size. This is commonly associated with the obliteration of small airways, which is presumed to be a result of pro-fibrinogenic substances secreted by NE cells. Clinically, the disease manifests with symptoms of constrictive bronchiolitis/small airway disease, including chronic cough and shortness of breath, which in some patients may for years be misinterpreted as symptoms of allergies or asthma. The pathogenesis of this disease is still poorly understood. Interestingly, it occurs almost exclusively in women. Radiology is highly distinctive and high-resolution CT scans are virtually pathognomonic, manifesting as multiple small punctate nodules, mosaic attenuation due to air trapping, and airway thickening^55,56^. Importantly, radiologically, DIPNECH may mimic the presentation of bilateral miliary metastases, particularly in patients on surveillance for known malignancies (like breast carcinoma)^55,56^.

There has been a recent debate in the literature on whether the term “DIPNECH” or “DIPNECH syndrome” should be restricted to clinically symptomatic patients^57^, in line with the original report on this condition by Aguayo et al. in 1992^58^. It is, however, recognized that there is a spectrum of clinical manifestations associated with this condition, ranging from severe to subtle and possibly subclinical, which may reflect the variable extent of the pathologic disease or other patient factors. In the latest WHO edition, the terms of pathologic DIPNECH and clinical DIPNECH are introduced^1^. Irrespective of the terminology used, it is important to document the presence of multiple tumorlets and NE hyperplasia, even if detected incidentally, to provide a correlate to potential detection of bilateral lesions on imaging.

Importantly, an isolated single tumorlet is an exceedingly common incidental finding and does not equate with the diagnosis of DIPNECH. These usually occur in the absence of multifocal intra-bronchial NE cell hyperplasia or obliterative bronchiolitis. Minimal quantitative criteria for DIPNECH in resections have been recently proposed as NE cell hyperplasia as 5+ NE cells in 3+ separate airways and at least 3 tumorlets^59^. It is also critical to distinguish DIPNECH—an extremely indolent condition from the oncologic perspective—from multifocal intrapulmonary carcinoid/NET metastases.

## Small cell carcinoma

### Morphologic spectrum

The prototypical morphology of small cell carcinoma is well recognized in various organs. The defining features of the classic, oat-cell morphology of SCLC include small cell size (<3 lymphocytes), finely dispersed chromatin without prominent nucleoli, scant cytoplasm with indistinct cell borders, and malleable/fragile nuclei, manifesting as molding, DNA streaming, and encrustation of vessels—the so-called Azzopardi effect^1,2,60^. Importantly, small cell size is not a strict criterion, and many SCLC have either subpopulations or overall cell sizes larger than 3 lymphocytes^2^. Extensive necrosis is characteristic, as is the high mitotic and apoptotic rate. Of note, although mitotic counts of >10 per 2 mm^2^ are listed as WHO criteria for SCLC, in practice, counting mitoses in obvious SCLC is usually not needed and in fact may not be feasible given the common presence of extensive karyorrhexis, degeneration, and low cell viability.

**Fig. 3:**
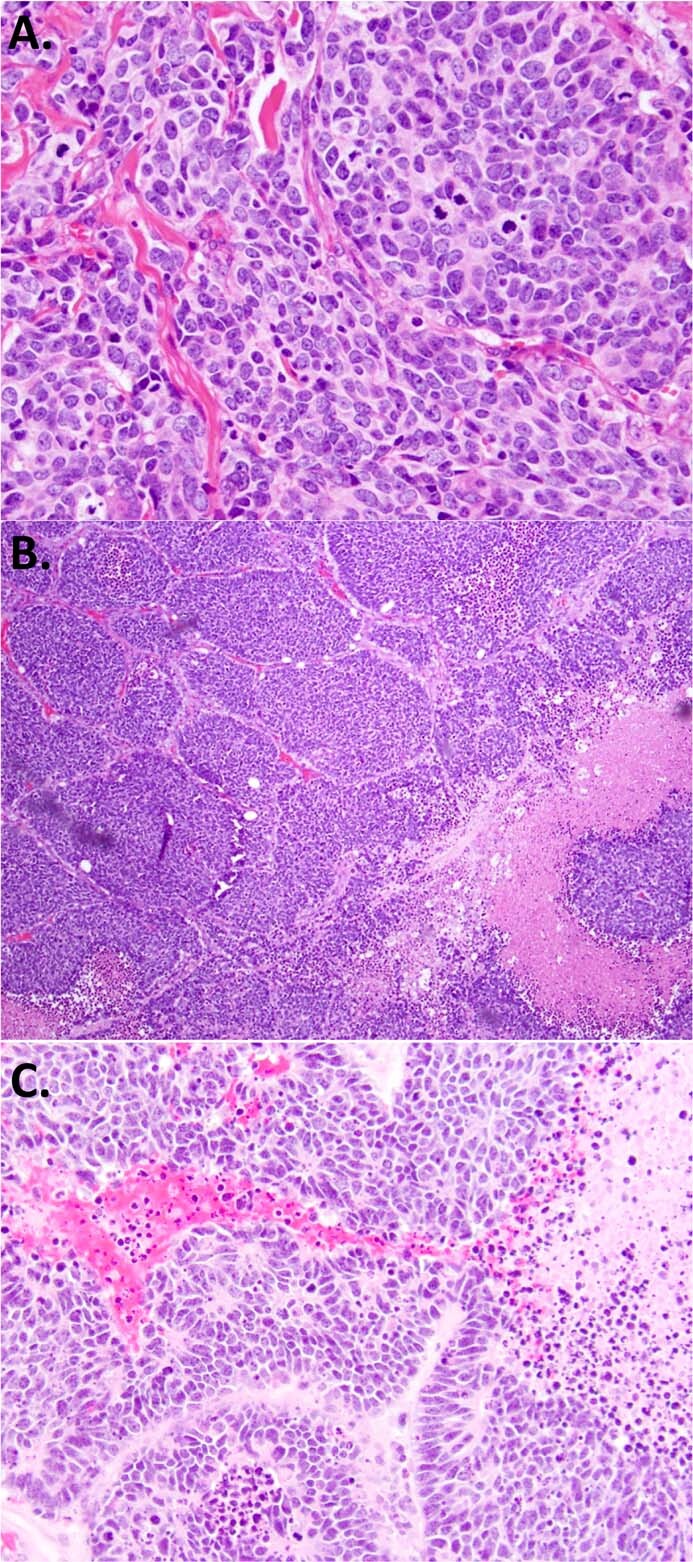
Morphologic features in SCLC that may be misinterpreted as LCNEC. **A** SCLC with larger overall cell size and more abundant cytoplasm, but otherwise morphologic features fitting the diagnosis of SCLC (notice there are no visible intercellular borders). **B** SCLC commonly have at least focal nested architecture; nested pattern alone does not favor the diagnosis of LCNEC over SCLC. **C** Rosettes are also common in SCLC.

Several variant morphologic features of SCLC are well-recognized in the literature but can appear unexpected in practice, causing diagnostic difficulties. Of these, the most important to recognize is SCLC variant formerly known as “intermediate-type” SCLC^2^. These tumors have an overall or focal areas with larger nuclear size, less cell crowding, and more visible cytoplasm (though no visible cell borders) (Fig. 3A). Because of similar clinical behavior, these SCLC are no longer recognized as a distinct type in WHO, but it is useful to be aware of this variant morphology in SCLC, particularly in considering the differential diagnosis with LCNEC. It is also critical to be aware that while most SCLC have diffuse/sheet-like pattern, SCLC can also have a nested/organoid architecture (Fig. 3B)^2^. In fact, in a study of surgically resected SCLC, 94% of cases had at least a focal nested pattern^61^. Rosettes—usually focal—are common in SCLC (Fig. 3C).

### Emerging biological subtypes of SCLC

Unlike NSCLC, SCLC is a fairly homogenous disease genomically, characterized by nearly invariable *RB1* and *TP53* alterations^62^. Multiple recent studies have identified that despite genomic homogeneity, SCLC comprises distinct subtypes based on the global differences in gene expression and methylation profiles. These subtypes are defined primarily by master transcriptional regulators (lineage factors): achaete-scute homolog 1 like (ASCL1), neurogenic differentiation factor 1 (NEUROD1), and POU class 2 homeobox 3 (POU2F3), with ASCL1-dominant SCLC representing the most common SCLC type (~70% of cases)^63,64^. ASCL1-dominant SCLC is thought to define the classic, chemosensitive type of SCLC, whereas other subtypes have been referred to as “variant” SCLC, associated with lower chemosensitivity^63^. Notably, ASCL1^+^ and NEUROD1^+^ SCLC are associated with the high level of NE marker expression (NE-high SCLC), whereas other subtypes, particularly POU2F3^+^, represent NE-low/negative SCLC (more on this below)^63,64^. ASCL1 and NEUROD1 are neurogenic master regulators, thought to regulate early (stem) vs late stages of differentiation, respectively^65^, while POU2F3 is a marker of chemosensory tuft cell lineage^66^. In recent studies, ASCL1/NEUROD1/POU2F3 triple-negative SCLC were found to be associated with mesenchymal transition and immune-related gene expression, designated as the fourth (SCLC-I) subtype. The role of YAP1, suggested as another subtype-defining marker in SCLC, requires further clarification^64,67^. In preclinical studies, these lineage factor-defined subtypes are associated with distinct therapeutics vulnerabilities, and it is anticipated that their identification may help guide the selection of therapy for patients with SCLC in the future clinical practice.

### The evolving role of IHC in the diagnosis of SCLC: a paradigm shift

Traditionally, small cell carcinoma (of lung and other-site origin) has been regarded as a morphologic diagnosis. Indeed, in well-preserved specimens where cytologic features can be adequately evaluated, and especially if there is at least focal evidence of rosettes, the diagnosis can be made based on morphology alone. However, confirmatory IHC has been increasingly utilized in practice to support the diagnosis of SCLC^1^, and in recent studies, it has been suggested that IHC increases the accuracy of SCLC diagnosis^68^. This represents a major paradigm shift in how SCLC is diagnosed. However, currently, there are no standard guidelines for which IHC markers represent a practical panel to support the diagnosis of SCLC. In the current thoracic WHO classification, IHC is stated as not required for the diagnosis of SCLC, but rather recommended as helpful for excluding an alternative diagnosis.

Generally, expression of NE markers is regarded as the main supporting evidence for SCLC diagnosis. However, it is well recognized that the expression of NE markers is highly variable in SCLC. As mentioned above, ~15–20% of SCLC are negative for the 2 most widely used NE markers—synaptophysin and chromogranin A. However, most of such cases are positive for CD56 and/or INSM1^13–15,69^. Nevertheless, even with a 4 NE marker panel, entirely NE-negative SCLC may still occur^15,69^, plus some SCLC have only minimal NE marker labeling (e.g., CD56-only in rare cells)^69^. TTF-1 is positive in the majority of SCLC (~80%), but it is usually associated with high NE marker expression^64^. Thus, cases that have low/negative expression of NE markers are also usually negative for TTF-1. In cases where the diagnosis of SCLC is favored morphologically, but all NE markers and TTF-1 are negative, the diagnosis can still be made provided that potential mimickers (such as lymphoma or basaloid squamous cell carcinoma) are excluded by other markers (such as CD45 and p40, respectively). In addition, recent data suggest that POU2F3 can provide additional support for the diagnosis of NE-low/negative SCLC.

### POU2F3: a new marker for NE-low/negative SCLC

POU2F3 (also known as SKN-1a/OCT-11) has been recently identified as a novel marker expressed in and essential for the viability of ~10–12% of SCLC, which is strongly associated with tumors that have low or negative expression of the standard NE markers^64,66^.

POU2F3 is a master regulator of the so-called tuft cells—named after the distinctive tuft-like apical microvilli. These are poorly understood cells scattered in various luminal surfaces throughout the body, including the airways. They are thought at least in part to have a chemosensory function, acting as “luminal sensors”^70,71^. Notably, POU2F3-positive SCLC exhibit a full gene expression program of tuft cells, and therefore have also been designated tuft cell-like SCLC^66^. Whether tuft-cell-like expression reflects a distinct cell of origin or a line of differentiation is currently unclear^64^. At the morphologic level, POU2F3^+^ SCLC appear to have typical characteristics of SCLC, but show enrichment in combined histology^64^.

**Fig. 4:**
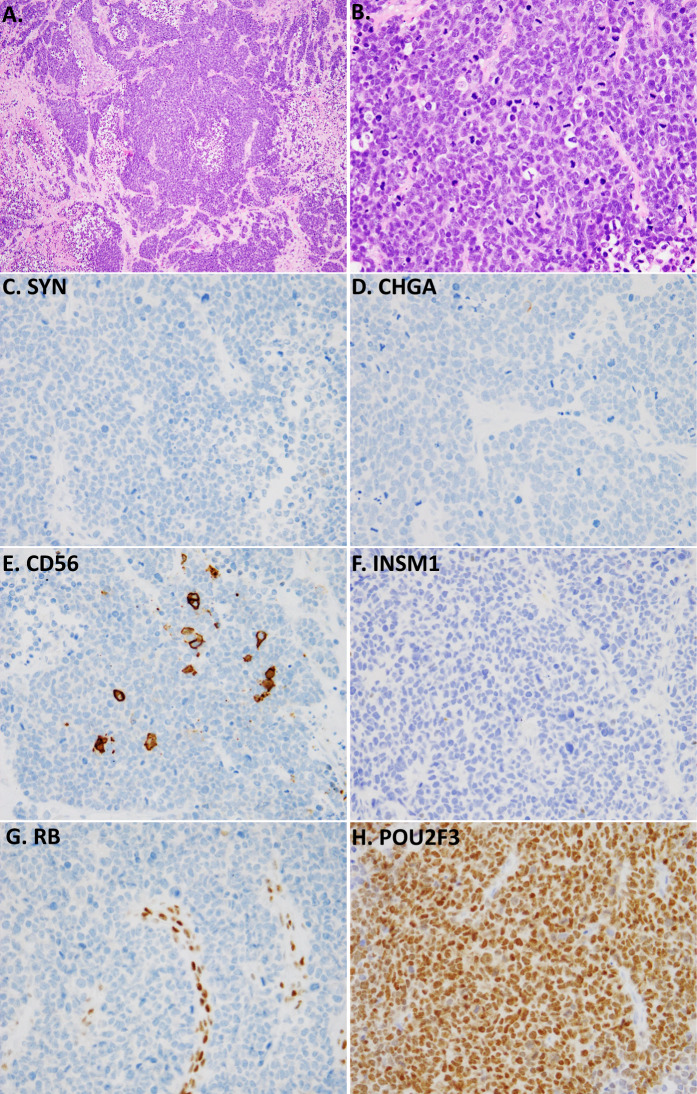
POU2F3 expression in SCLC with minimal reactivity for standard NE markers. Low-power (**A**) and high-power (**B**) images showing morphologic appearance suggestive of SCLC, but with completely negative labeling for synaptophysin (SYN; **C**), chromogranin A (CHGA, **D**), and INSM1 (**F**). Only CD56—the least specific NE marker—shows labeling in scattered cells (**E**). The diagnosis of SCLC is supported by the strong expression of POU2F3. As expected for small cell carcinoma, Rb shows the loss of expression in tumor cells relative to entrapped benign cells (**G**). Photo credit: Marina K Baine.

In recent studies, our group found that POU2F3 can serve as a helpful diagnostic marker in clinical practice^69^. Namely, we analyzed SCLC (or suspected SCLC) in which labeling for all 4 NE markers was either completely negative or minimal. Although uncommon, when encountered, these tumors can present a diagnostic challenge (Fig. 4). We found that POU2F3 was robustly expressed in the majority of such cases, suggesting that it may serve as a helpful marker in practice. Although the diagnosis of NE-negative/minimal SCLC can be made without POU2F3, this may require performing a set of markers to exclude various mimickers, whereas a positive diagnostic marker could allow for a more efficient work-up.

### Update on the differential diagnosis for SCLC (and LCNEC)

A critical consideration in the diagnosis of SCLC (and LCNEC) is the fact that various NSCLC can express NE markers. Overall, ~10–20% of conventional adenocarcinoma and squamous cell carcinoma (SqCC) can exhibit NE marker labeling^1,2,12^. Therefore, the diagnosis of NECs should only be made in the context of appropriate morphology (+/− other markers), and should not rely on NE marker positivity alone, especially if focal and limited to a single marker.

**Fig. 5:**
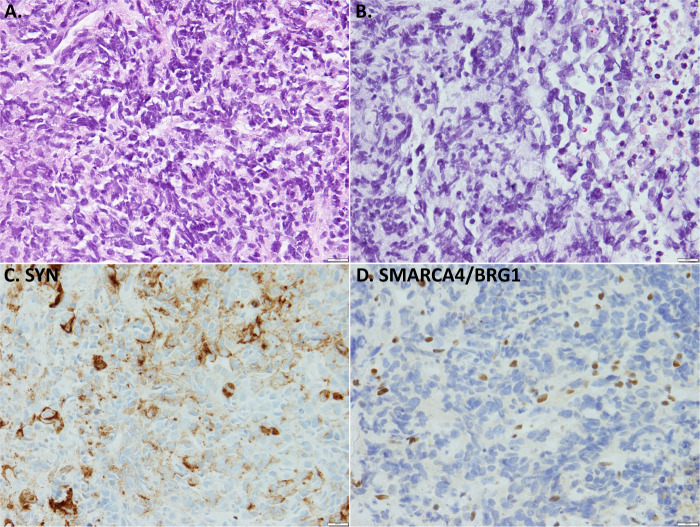
Thoracic SMARCA4-deficient undifferentiated tumor as a mimicker of SCLC. Low-power (**A**) and higher-power (**B**) histologic images of highly necrotic round cell tumor with crush artifact and molding, which in conjunction with synaptophysin labeling (SYN, **C**) closely mimics SCLC. In better-preserved areas, prominent nucleoli and rhabdoid cells were seen (not shown). The diagnosis is confirmed by the loss of SMARCA4 (BRG1) expression in tumor cells relative to entrapped benign cells (**D**). Other markers supporting the diagnosis include SMARCA2 (BRM) co-deficiency and the expression of stem-cell markers (SALL4, CD34).

Of note, there are several entities entering in the differential diagnosis with SCLC/LCNEC that are particularly prone to the expression of NE markers. This most notably includes the recently defined entity of thoracic SMARCA4-deficient undifferentiated tumors (SMARCA4-UT)^72–74^. In prior studies, >70% of these tumors have been reported to label for synaptophysin, commonly diffusely^72^. These tumors can clinically mimic NECs in that they typically occur in heavy smokers, and present as large central locally infiltrative masses with extensive metastases. Morphologically, these are high-grade tumors that prototypically have focal to extensive rhabdoid morphology, but some cases have undifferentiated round cell morphology. These tumors do not display NE morphology (no rosettes, no nests/palisading), but when extensively necrotic and affected by crush artifact in biopsies, combined with synaptophysin positivity and extremely high Ki67, they can closely mimic NECs (Fig. 5). In fact, in a study from our group, nearly a quarter of SMARCA4-UT were initially diagnosed as small-cell or large-cell NECs^72^. These tumor have a distinctive IHC profile (loss of SMARCA4/BRG1, loss of SMARCA2/BRM, and expression of stem-cell markers SALL4, CD34, SOX2), although SOX2 is also commonly positive in NECs. It is important to be aware of this potential close mimicker in the differential diagnosis with NECs, given that SMARCA4-UT are highly chemoresistant and specific therapeutic strategies to target tumors with SWI/SNF complex deficiencies are under development.

Another close mimicker of SCLC in smokers is basaloid SqCC. These tumors can exhibit remarkable morphologic similarity to SCLC (or LCNEC), especially when extensively necrotic^1,60^. Presence of matrix deposition is a clue to the diagnosis, but this feature can be present only focally. Thus, in NE-negative carcinomas where the diagnosis of SCLC is being considered, adding p40/p63 to exclude the possibility of basaloid SqCC is an important consideration. Of note, CD56 can be occasionally diffusely positive in basaloid SqCC of various sites, including lung^1,75^.

In never/light smokers, tumors with the appearance suggestive of SCLC should be diagnosed as such with extreme caution, and only after various other mimickers are firmly excluded. Most commonly this includes biopsies of crushed carcinoids, where the Ki67 index generally readily resolves this differential, as discussed above. Other considerations that can be readily resolved by IHC (and if needed FISH/molecular studies) include lymphomas and various round cell sarcomas (including Ewing sarcoma, which can be positive for NE markers). NUT carcinoma is another extremely rare potential mimicker of NECs; recent studies have documented the possibility of significant NE marker expression in these tumors^76,77^. Lastly, Merkel cell carcinomas (MCC) are NE carcinomas that are morphologically similar to SCLC. By IHC, they are negative for TTF-1 and positive for Merkel cell polyomavirus (MCPyV), CK20 (dot-like) and neurofilament (dot-like). In practice, a question is occasionally raised whether the possibility of an occult MCC should be routinely ruled out in all specimens from suspected SCLC. It should be remembered, however, that clinical presentation of MCC is highly distinctive from SCLC—it is a notoriously nodotropic disease, and lung involvement in the absence of peripheral adenopathy would be an extremely unusual presentation for this tumor^78^.

If a SCLC diagnosis is confirmed in a never-smoker, the differential diagnosis may include a limited sampling of a combined carcinoma, such as combined SCLC and adenocarcinoma that may harbor *EGFR* mutations^79^. Lastly, SCLC in a never-smoker may represent an unsuspected metastasis, such as from HPV-related sites like cervix or oropharynx, and which can be confirmed by ancillary studies for HPV.

### Small cell transformation as a form of acquired resistance to targeted therapies for lung adenocarcinoma

Transformation to SCLC (and occasionally LCNEC) occurs in ~5–14% of patients receiving targeted therapies for *EGFR-*mutated lung adenocarcinomas. This has been documented for the first (erlotinib), second (afatinib), and third (osimertinib) generation EGFR inhibitors^80–82^. Small cell transformation has also been documented with ALK inhibitors^83^. NEC transformation appears to be a general mechanism of evasion of targeted tumor inhibition, as also well documented for prostate carcinoma undergoing androgen deprivation therapy^84^. Small cell transformation is critical to document since this has a significant impact on clinical management.

Is it possible to identify which tumors that are likely to transform on therapy? Indeed, studies show that *EGFR*-mutated adenocarcinomas that harbor *RB1* and *TP53* alterations at baseline are uniquely predisposed to transformation, as virtually all transformed tumors have these mutations prior to therapy^81^. The role of *RB1* and *TP53* testing in *EGFR*-mutated adenocarcinoma, and the use of more aggressive therapy upfront in tumors harboring these alterations is currently under investigation (https://clinicaltrials.gov/ct2/show/NCT03567642).

### Potential predictive markers in SCLC

Currently, there are no standard biomarkers used to guide clinical decisions in SCLC. While there is encouraging data on the potential of using transcriptional subtype markers (ASCL1, NEUROD1, POU2F3) for therapy selection in SCLC, this remains investigational at the time of writing. PD-L1 is rarely expressed in SCLC^85^, and the recent approval of first-line immunotherapy for SCLC does not require evaluation of PD-L1^62^.

Delta-like canonical Notch ligand 3 (DLL3) has attracted recent attention as a molecule that is highly expressed in SCLC, and as a therapeutic target of antibody–drug conjugate rovalpituzumab tesirine (ROVA-T). Despite initial encouraging results in SCLC, the development of ROVA-T was suspended after subsequent studies^86,87^. Nevertheless, several other clinical trials with DLL3 as a therapeutic target are ongoing^86,87^. DLL3 can be readily detected by IHC in SCLC (~80%)^64,88^ and LCNEC (~75%)^89^. Expression of DLL3 is strongly linked with ASCL1^+^ or NEUROD1^+^ SCLC subtypes^64^. Its potential role as a biomarker awaits the results of the ongoing clinical trials.

## Large cell neuroendocrine carcinoma

### WHO definition

The definition and the approach to LCNEC remain largely unchanged in 2021 WHO classification from the prior edition, with exception of the clarification regarding the feasibility of the diagnosis in biopsies. LCNEC is defined as tumors with NE morphology (organoid nesting with palisading, trabeculae, rosettes), cytologic features of non-small cell carcinoma (large cells size, prominent nucleoli, and/or abundant cytoplasm), and high proliferation rate, defined as >10 mitoses per 2 mm^2^, but generally substantially exceeding this threshold (median 70 mitoses/2 mm^2^). Expression of at least one of the 3 standard NE markers (synaptophysin, chromogranin A, CD56) is required for the diagnosis. Notably, there is no minimal requirement for the extent of NE marker expression, provided that NE morphology is convincing^1^. However, caution should be exercised when the diagnosis is considered for tumors labeling for CD56 alone, given that this is the least specific NE marker, even if diffuse. The role of recent NE markers (INSM1, ASCL1) in the diagnosis of LCNEC remains to be clarified.

Ki67 is not part of the WHO criteria for the diagnosis of LCNEC, but as mentioned above, it may be helpful in the distinction of LCNEC from carcinoids in biopsies, especially those with a crush artifact. Of note, Ki67 ranges in LCNEC are significantly broader than those for SCLC. While many cases have a Ki67 rate of 70–100%, some LCNECs have lower values, overlapping with those in the upper zone of highly proliferative carcinoids. Thus, Ki67 gray zone of 20–60% particularly applies to LCNEC^22,90^.

### Morphologic spectrum in LCNEC

**Fig. 6:**
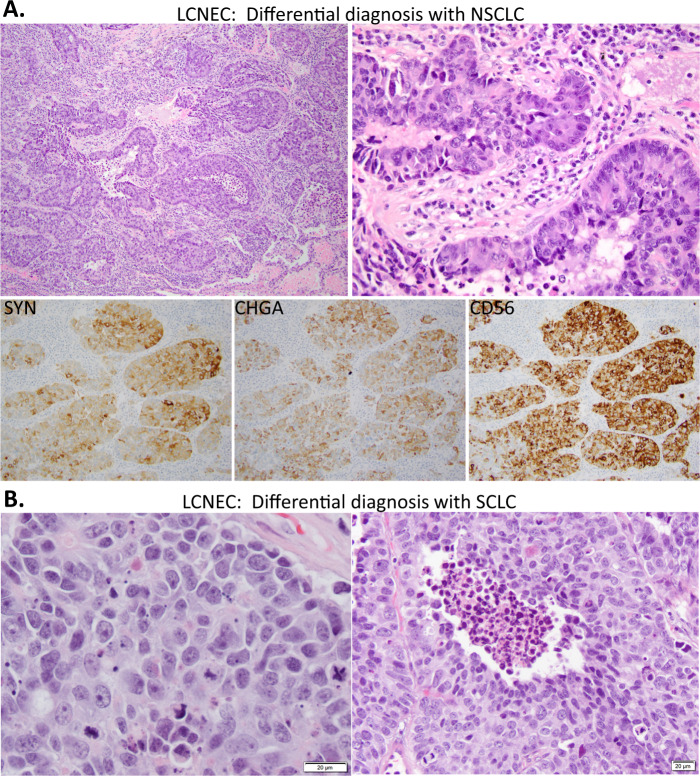
Prototypical morphologic spectrum in LCNEC. Panel **A** illustrates LCNEC morphology falling in the differential diagnosis with NSCLC. Basaloid appearance, with rosettes and palisading, together with robust expression of NE markers supports the diagnosis. Panel **B** illustrates two LCNECs with morphology falling in the differential diagnosis with SCLC—cells are more crowded with molding but show obvious nucleoli and visible intercellular membranes.

The morphologic spectrum in LCNEC generally spans two relatively distinct extremes. On the one end are tumors that have clear-cut non-small cell cytology (large cells, abundant cytoplasm, prominent nucleoli) but distinctly NE architecture—nesting with palisading, trabeculae, rosettes—the patterns seen in prototypical NE and neuroblastic tumors (Fig. 6A). Chromatin in most cases is coarsely granular but can be vesicular. Such tumors commonly have a low-power basaloid appearance with anastomosing nests/trabeculae, and cytoplasm may have amphophilic quality. In tumors with this morphology, SCLC is usually not a significant differential diagnostic consideration. Here, the differential diagnosis is instead with NSCLC, including adenocarcinomas with solid/cribriform pattern, large cell carcinoma, or basaloid SqCC. In our practice at MSKCC, this is the most common morphologic appearance of LCNEC. Yet, this type of LCNEC may be under-recognized in practice as the above entities. Although treatment implications are still being defined, the recognition of LCNEC as separate from poorly differentiated NSCLC is justified, at least in part, by the association with extremely poor prognosis and high rate of brain metastases.

At the other end of the morphologic spectrum are tumors that do enter in the differential diagnosis with SCLC (Fig. 6B). LCNECs of this type typically have nuclear features that overall resemble SCLC (small or intermediate cell size, coarsely granular chromatin) but which have either more prominent nucleoli than typical of SCLC or more abundant cytoplasm. Visibility of intercellular membranes is a useful criterion to support the diagnosis of LCNEC since scant cytoplasm in SCLC leads to consistently inapparent intercellular borders^1,91^. This is the morphologic differential that can suffer from significant inter-observer disagreement, even among experienced pulmonary pathologists^92,93^.

Third, LCNEC may have morphologic resemblance to atypical carcinoids. In most cases, tumors with the morphology of carcinoids (bland cytology, smooth nuclear membranes, absent or at most focal/punctate necrosis) but mitotic counts mildly exceeding 10 per 2 mm^2^ represent what is increasingly recognized as highly proliferative carcinoids, analogous to digestive grade 3 NETs.

### Recent molecular insights: two major types of LCNEC and potential clinical implications

Several recent molecular studies have identified two major types of pulmonary LCNEC—one with genomic features of SCLC, characterized by *RB1* and *TP53* alterations, and the other with genomic features of NSCLC, characterized primarily by alterations typical of smoking-associated adenocarcinoma (*STK11*, *KEAP1*, *KRAS*) and the absence of *RB1* alterations, with a minority of cases exhibiting mixed or indeterminate features^22,94,95^.

Overall, these molecular findings support the concept of LCNEC representing a “mixed bag” of tumors of different origin—one related to SCLC and the other to NSCLC. In fact, the “large cell” morphology is long known to occur in SCLC cell lines^96^, and LCNEC subpopulations are commonly present in mouse models of SCLC arising from NE precursors^97^, which has been associated with aggressive and treatment-resistant behavior^96^. Conversely, NSCLC-subtype of LCNEC may be histogenetically related to lung adenocarcinomas harboring *STK11*/*KEAP1*/*KRAS* mutations—a subset that is also known to be associated with chemoresistance and aggressive behavior^98^. Thus, LCNEC may represent a convergence of aggressive and chemoresistant variants of both SCLC and NSCLC.

Do molecular subtypes simply reflect the spectrum of morphology in LCNEC? Indeed, in a study from our group, there was significant correspondence between LCNEC molecular subtype and morphology, such that tumors with SCLC-type genomics exhibited greater cell crowding and higher proliferation rates than NSCLC-type LCNECs^22^. However, some cases had indeterminate morphology, emphasizing that this should not be regarded as an entirely morphologic distinction^22^.

Historically, the treatment approach to LCNEC has been a matter of a long-standing uncertainty, with both SCLC-type and NSCLC-type systemic therapies used in practice^99,100^. Recently, several retrospective clinical studies have found differences in prognosis and treatment outcomes of LCNEC patients based on molecular subtype, as determined primarily by the status of Rb (by molecular testing and/or IHC)^101,102^. Nevertheless, even Rb-deficient LCNEC appear to have lower chemosensitivity than conventional SCLC^102^, emphasizing the need for novel treatment approaches for these tumors.

Future studies are much needed to firmly establish the utility and method of identifying subtypes of LCNEC for guiding treatment decisions in routine clinical practice. In addition to Rb, Ki67 may also have a role, given that LCNEC with SCLC-type genomics are usually associated with the higher Ki67 rates (>70%)^22^. Also, interestingly, in a recent study by Milione et al., Ki67 of >55% was found to be a strong predictor of unfavorable prognosis in LCNEC^90^, analogous to the findings in digestive NECs^103^. Provisionally, in investigational setting, LCNEC that exhibit Rb mutations/loss, particularly when associated with extremely high Ki67 rates (70–100%), could be regarded as likely representing a SCLC-related variant of LCNEC, and patients could be stratified accordingly for clinical outcomes analysis; studies of this type are currently in progress^104^.

Lastly, targetable genomic alterations typical of adenocarcinoma do occur in LCNEC (NSCLC-type). Therefore, LCNEC should be handled with “NSCLC-type” molecular testing protocols^1,22^. While alterations like *EGFR* and *ALK* are rare in LCNEC since these are primarily smoking-associated tumors, *KRAS* G12C mutations have a similar prevalence to that in adenocarcinoma (~13%)^22^. Therefore, molecular testing is an important consideration in LCNEC given the recent development of effective targeted agents for *KRAS* G12C^105^.

### A practical approach to the differential diagnosis of LCNEC

The main differential of LCNEC with NSCLC and SCLC is highlighted here; for detailed review of the topic please see the recent article by Baine and Rekhtman^91^. Given the emerging understanding of LCNEC as a mix of tumors molecularly related to NSCLC and SCLC, the major differential diagnosis of LCNEC can be understood in the context of these relationships. Thus, some diagnostic challenges may represent a true biologic continuum between LCNEC and NSCLC on the one end, and LCNEC and SCLC on the other end.

#### LCNEC vs NSCLC

Given the known expression of NE markers in a subset of NSCLC, such expression in isolation does not equal LCNEC. Nevertheless, most LCNECs (>80%) are positive for 2–3 standard NE markers (synaptophysin, chromogranin A, CD56), whereas most conventional NSCLC usually label for only 1 marker, typically only focally, with labeling for 2+ markers reported in only 1–4% of cases^91,106^. However, as mentioned above, some poorly differentiated tumors (like SMARCA4-UT) are particularly prone to NE marker expression. Thus, in isolation, NE marker expression should not be used as the sole basis for LCNEC diagnosis.

Other than NE markers—with the given caveats—there are no other IHC markers that can aid in this differential diagnosis with high sensitivity and specificity. Strong, diffuse expression of exocrine marker Napsin A favors adenocarcinoma, but some LCNECs (~15%) can express this marker, although usually only weakly/focally^90,107,108^. Overall, TTF-1-diffuse/Napsin A-negative (or weak) profile should raise a consideration of NEC (small cell or large cell) since most adenocarcinomas with robust TTF-1 expression are also strongly positive for Napsin A, but this is not invariable.

Although much of reproducibility studies in LCNEC have focused on challenges with SCLC differential, there can also be notable subjectivity in the differential of solid/cribriform adenocarcinoma or large cell carcinoma vs LCNEC since NE morphology can range from robust to subtle. Challenges can arise for cases with equivocal/borderline NE morphology (e.g., cribriform spaces vs rosettes) and weak/focal NE marker expression, and this area would greatly benefit from development of additional diagnostic markers.

#### LCNEC vs SCLC

This is largely an entirely morphologic distinction based on a constellation of features (cell size, prominent nucleoli, abundant cytoplasm such that intercellular membranes are visible). It is in this differential diagnosis that the issue of the spectrum of morphology in SCLC comes into play. Importantly, as discussed in the section on SCLC, simply the presence of nested architecture does not argue against the diagnosis of SCLC. Similarly, the larger overall cell size with otherwise typical nuclear features and more spaced out nuclei but in the absence of visible cell membranes can be in line with “intermediate type” morphology in SCLC rather than LCNEC.

Yet, in a subset of cases, morphologic features can have borderline characteristics, and the distinction can be further limited by crush artifact or extensive necrosis in biopsies. In some cases, the availability of a high-quality H&E stain can significantly aid in the evaluation of tumors in this differential, as can the availably of cytologic preparations where the crush artifact tends to be minimized. Importantly, the presence of any amount of convincing SCLC morphology qualifies a tumor that is predominantly LCNEC for the diagnosis of combined SCLC and LCNEC.

Although there are no sensitive and specific markers for LCNEC vs SCLC diagnosis, any reactivity for Napsin A would favor the former as this marker is consistently negative in SCLC, but sensitivity of this is very low^107^. Dot-like reactivity for keratins is regarded as a hallmark of SCLC, but can also be seen in LCNEC^12,91^. Lastly, retained Rb expression is more common in LCNEC than SCLC but this is not entirely specific^109^.

As with the differential of NSCLC, the differential of LCNEC vs SCLC would greatly benefit from the development of objective diagnostic markers that pathologist could utilize in routine practice.

#### LCNEC vs other

A variety of other poorly differentiated lung and metastatic tumors may enter in the differential diagnosis with LCNEC, most notably SMARCA4-UT and basaloid SqCC, as discussed for SCLC^91^.

### Update on the diagnosis of LCNEC in biopsies and cytology

Previously, the diagnosis of LCNEC in biopsies and FNA has been discouraged^110^. Indeed, given that the diagnosis of LCNEC relies to a great extent on the architectural pattern in the context of poorly differentiated and frequently markedly necrotic carcinomas, the diagnosis may be difficult in small, disrupted, or poorly preserved specimens. However, with the recent trend to obtain larger volumes of tissue in patients with lung tumors for potential molecular testing, the diagnosis of LCNEC in biopsies has become feasible more often in recent years^1^.

**Fig. 7:**
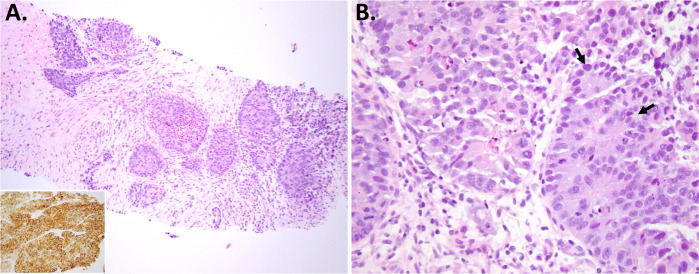
Example of LCNEC diagnosis in a core biopsy. Low-power (**A**) and high-power (**B**) images show a clear-cut NSCLC but with NE architecture (trabeculae, palisaded nests, rosettes), with high proliferation rate, extensive necrosis, and diffuse expression of NE markers (SYN shown in inset in **A**). Biopsy of this type can be diagnosed as definite LCNEC. Arrows in **B** mark rosettes.

Figure 7 illustrates a definitive diagnosis of LCNEC in a core biopsy, which is supported by evident NE architecture, clear non-small cell cytology, high mitotic rate, presence of significant necrosis, and expression of NE markers. Recent studies show that NE morphology is apparent in most (50–85%) but not all biopsies from subsequently resected LCNEC^108,111^, in part depending on the biopsy size^111^.

In a recent study, Baine et al. proposed a semiquantitative score for distinguishing LCNEC from NSCLC in biopsies^108^. As such, tumors were assigned 1 point for each morphologic feature of NE differentiation (palisading, rosettes, nesting), 1 for the presence of necrosis, 1 for Ki67 of ≥40%, and 3 for NE marker positivity (staining with any extent for any one or more NE marker). The diagnosis of LCNEC is supported by a score of 4 or higher with high sensitivity and specificity. At least 1 point should come from NE morphology and staining for at least one NE marker is required, based on LCNEC criteria.

It was also suggested that positivity for 2 or more NE markers in a biopsy of NSCLC with no morphologic evidence of adenocarcinoma or SqCC would qualify for the diagnosis of LCNEC^111^. Indeed, the odds of 2 NE marker expression, especially if robust, is much higher for LCNEC than other NSCLCs. Nevertheless, given even a small possibility of such expression in non-NE tumors, as discussed above, the diagnosis of LCNEC should only be suggested in the context of the appropriate morphology.

In biopsies where the diagnosis of LCNEC is considered but either there is insufficient material for IHC, equivocal morphologic features or poor tissue preservation, depending on the spectrum of findings, according to the current WHO, the diagnosis could be rendered as “NSCLC, possible LCNEC” or “NEC, not otherwise specified”^1,91^.

In cytology samples, the definitive diagnosis of LCNEC can be difficult, but it may be suspected in cases with cellular cell blocks that allow for the assessment of NE architecture and IHC^1^. In smears, cytologic features of LCNEC can resemble SCLC or NSCLC, analogous to histologic specimens^1^.

## Combined neuroendocrine carcinomas

Combined NE carcinomas are defined by the WHO as tumors containing both SCLC or LCNEC components plus any type of NSCLC component (adenocarcinoma, SqCC, large cell carcinoma, rarely other). For combined SCLC, the most common combination is with LCNEC or large cell carcinoma (~10% of cases), whereas combination with other NSCLC components is uncommon (3–9%)^61,62,64^. Given the common presence of occasional large cells in SCLC, only if they exceed 10% is it recommended by the WHO to classify as combined carcinoma. For adenocarcinoma or SqCC, any amount of such component qualifies for combined SCLC diagnosis^1^.

Carcinomas with co-existing NE and mucinous differentiation in the same cell rather than in distinct geographic areas (so-called amphicrine carcinomas) do rarely occur in the lung^112^, although they are better described in the digestive tract^113^. The WHO taxonomy for these is currently lacking, but conceptually such tumor may be regarded as a type of combined carcinomas.

Recent multi-omic molecular studies on combined SCLC have started to provide first insights into molecular mechanisms underlying the fascinating lineage plasticity in these tumors^114^.

Unlike carcinomas, carcinoids generally do not occur in combination with NSCLC, supporting their highly distinct histogenesis, with only rare exceptions documented in case reports^115^.

## NSCLC with isolated neuroendocrine morphology or neuroendocrine marker expression

The term “large cell carcinoma with NE morphology” refers to rare tumors that have LCNEC morphology but lack demonstrable neuroendocrine marker expression by IHC. Clinical data on such tumors are scarce but have suggested aggressive behavior similar to that of LCNEC^1^. Future studies will be needed to reassess these tumors in the context of the recent NE markers (INSM1, ASCL1) and chemosensory marker POU2F3.

The term “NSCLC with NE differentiation” refers to conventional NSCLC (adenocarcinoma, SqCC, large cell carcinoma, etc) that do not exhibit NE morphology but do express NE marker(s). Given the lack of consistent data to support clinical relevance, staining for NE markers in the absence of NE morphology is not recommended^1^.

## The role of molecular testing and PD-L1 in lung NE neoplasms

Unlike NSCLC, there are no established molecular therapeutic targets in lung carcinoids and SCLC, and molecular testing of these tumors outside of investigational protocols is not currently recommended. Conversely, LCNEC (particularly if known to have retained Rb expression) should be tested using the approaches established for NSCLC. Also, unlike NSCLC, there is no established role of PD-L1 testing in lung carcinoids, SCLC or LCNEC.

## Conclusions

This article has summarized key recent developments relevant to the pathologic diagnosis and biological understanding of lung neuroendocrine neoplasms. There has been major progress in elucidating genomic makeup and biological subtypes within each major type of NE neoplasm. Nevertheless, there are several persistent challenges in pathologic diagnosis, that would significantly benefit from the development of diagnostic markers. It is hoped that the recent molecular progress will yield novel diagnostic, prognostic, and predictive tools, as well as serve as a basis for novel treatment approaches in the near future.

## Data Availability

Not applicable.
